# The Costs of Climate Change: A Study of Cholera in Tanzania

**DOI:** 10.3390/ijerph8124386

**Published:** 2011-11-28

**Authors:** Sara L. M. Trærup, Ramon A. Ortiz, Anil Markandya

**Affiliations:** 1 UNEP Risø Centre, Risø DTU, P.O. Box 49, 4000 Roskilde, Denmark; 2 Basque Centre for Climate Change BC3, Gran Via, 35-2, Bilbao 48005, Spain; Email: ramon.ortiz@bc3research.org (R.A.O.); anil.markandya@bc3research.org (A.M.)

**Keywords:** climate change, health impacts, cholera, adaptation costs, Tanzania

## Abstract

Increased temperatures and changes in rainfall patterns as a result of climate change are widely recognized to entail potentially serious consequences for human health, including an increased risk of diarrheal diseases. This study integrates historical data on temperature and rainfall with the burden of disease from cholera in Tanzania and uses socioeconomic data to control for the impacts of general development on the risk of cholera. The results show a significant relationship between temperature and the incidence of cholera. For a 1 degree Celsius temperature increase the initial relative risk of cholera increases by 15 to 29 percent. Based on the modeling results, we project the number and costs of additional cases of cholera that can be attributed to climate change by 2030 in Tanzania for a 1 and 2 degree increase in temperatures, respectively. The total costs of cholera attributable to climate change are shown to be in the range of 0.32 to 1.4 percent of GDP in Tanzania 2030. The results provide useful insights into national-level estimates of the implications of climate change on the health sector and offer information which can feed into both national and international debates on financing and planning adaptation.

## 1. Introduction

With continuous and increasing rates of morbidity associated with waterborne diseases, especially in Sub-Saharan Africa [1], this group of diseases is potentially an significant economic burden, leading to high direct costs to the health sector and to households, and indirectly to the economy and society at large because of time lost allocated to work, school and other productive activities. For example, water-related diarrheal diseases, including cholera, are widespread in areas where water resources are scarce, the majority of such diseases being attributed to environmental factors such as unsafe drinking water, poor hygiene and lack of sanitation [2]. Subsequent work by various international experts, including climate modelers, has assessed the potential climate change impacts in different parts of Africa at a relatively detailed level [3]. Among the conclusions that emerge is an agreement across most models to the effect that future climate change in East Africa will produce a tendency to increased precipitation in the winter months , but that this may also be combined with an increased intensity of rain in shorter periods and drought in other periods.

There is increasing emphasis on quantifying the health impacts from climate change [4,5]. Recent studies have associated temperatures and rainfall anomalies with diarrhea and cholera, and stress the role of climate variability in cholera transmission in Africa [6,7,8,9]. Higher ambient temperatures lead to higher water temperatures in shallow bodies of water, such as ponds and rivers and shallow coastal waters, and a recent study [10] has shown that both an increase in local temperature and the occurrence of floods caused by heavy monsoons can contaminate drinking water and influence the prevalence of the disease. The World Health Organization (WHO) has examined the global burden of disease attributable to climate change up to 2000, concluding against this background that planning health adaptation to climate-change impacts will require detailed assessments of national vulnerabilities to specific health risks [11]. Additionally, the WHO projected the health burden for Sub-Saharan Africa in 2030. The projections are based on case studies from Peru [12] and Fiji [13], where the data from Peru showed an increase of 8 percent for every 1 degree increase in temperature, while the study from Fiji showed an increase of 3 percent per degree of temperature increase. Since then, the WHO has used an average of 5 percent to predict the increase in the relative number of diarrheal incidences for 2030, taking into account socio-economic development and increased coverage of water and sanitation [14]. 

This vulnerability to current climate variability and future climate change can be reduced by means of adaptation. Adaptation, broadly defined, would include all current and future activities or interventions that reduce or prevent additional cases of disease or deaths attributable to climate change [15]. Adaptation would include measures to reduce the acute and chronic health impacts of extreme events (heatwaves, floods and droughts). Several reviews of health adaptation strategies, policies and measures have now been published [15,16,17,18]. The projected climate change impacts for Sub-Saharan Africa indicate increased rainfall variability, increased temperatures and prolonged droughts [3]. Therefore, the magnitude of climate change-related health impacts over the next few decades will largely depend on the effectiveness and timing of adaptation measures. For this purpose, decision-makers and national governments need information on the extent of the damage attributable to climate change, the financial resources needed for adaptation, and on what types of damage can be avoided through proposed adaptation measures. According to the WHO, improvements in water supply and sanitation are the most sustainable approach for the prevention of water-related diarrheal diseases, including cholera [19]. A pressing challenge is therefore the need for improved water supply. A general main development objective is to increase access to clean, affordable and safe water and sanitation and to reduce vulnerability from environmental risks. 

There exist several reviews and studies of the costs of health intervention programmes which address the prevention of climate-related diarrhea and cholera [20,21,22,23,24]. These studies present measures to reduce vulnerability to current events. Other recent studies have turned to focusing specifically on adaptation costs, including those related to reducing the impacts of future climate change [25,26]. However, there is very little information in the literature on the costs of health adaptation, especially for developing countries and on a country level. This could be attributed to adaptation being a relatively new field and the difficulties in quantifying costs, especially in developing countries, due to a lack of data and long-term reporting on disease and climate variables. Nevertheless, the aim of this article is to provide new evidence for the impacts of climate change on the prevalence of cholera using local (national) original data in addition to estimating the costs of these impacts. This should be seen as complementary information to previous aggregated estimates of the burden of disease attributable to diarrheal diseases regionally for Sub-Saharan Africa, which were estimated based on data from a different region of the world.

Specifically, this article focuses on Tanzania, where several major cholera epidemics have occurred in recent decades [27]. The analysis draws on primary data sources to estimate the relationship between climate variables and cholera in Tanzania and uses these results for projections of the future burden of cholera attributable to climate change in the country by 2030. These results provide a basis for estimating the total costs of cholera that are attributable to climate change, including the costs of reactive adaptation, productivity losses and lost lives. The paper is organized as follows. The next section provides information on the data and methodology used in the quantification of the impacts and estimation of costs. This is followed by a section on the results. These results are discussed in the next section in the context of the implementation of preventive adaptation measures to reduce current and future vulnerabilities to climate-change impacts. Finally, the paper ends by laying out the main conclusions of the study.

## 2. Methodological Issues

### 2.1. Data

Historical data on deaths and cases of cholera were acquired from the Ministry of Health in Dar es Salaam. Two data sets were available. One data set covering cholera cases throughout the country on a monthly basis between January 1998 and December 2004, while a second data set for cholera cases and deaths were available on an annual basis for the whole of Tanzania (1977 to 2004), as well as for 21 of its regions (1998 to 2004). The climate variables were acquired from the Tanzania Meteorological Agency, and include rainfall (monthly totals in millimeters) and temperatures (monthly mean of daily minimum and maximum temperatures). The variables represent the average of 19 weather stations throughout the country. In addition to climate and health variables, socioeconomic data [28,29,30,31,32,33] were gathered for the datasets containing data per year at the country level only. Therefore, three datasets were created for the purpose of the analysis presented here, as follows:

**Table 1:** Overview of datasets.

Time aggregation	Geographical aggregation	Health Endpoints	Climatic and socio-demographic variables
Months (1998–2004)	Country level	Cholera cases	Rainfall (millimeters); average min. and max. temperatures (°C); dummy representing the drought season
Year (1977–2004)	Country level	Cholera cases and fatalities	Rainfall (millimeters); average min. and max. temperatures (°C); real GDP/capita (US$); water and sanitation cover (% of households); population and cassava production (tons)
Year (1998–2004)	Regional level (21 regions)	Cholera cases and fatalities	Rainfall (millimeters); average min. and max. temperatures (°C)

Note: The panel dataset was formed from data on 21 units (regions) for 7 years.

**Figure 1:**
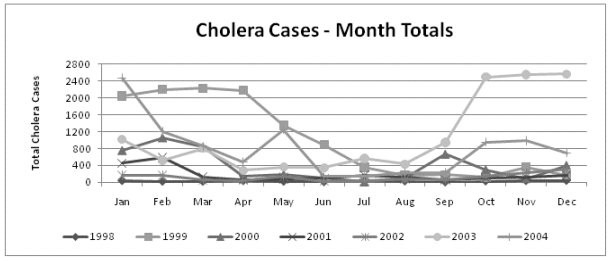
Seasonal distribution of cholera cases in Tanzania.

**Figure 2:**
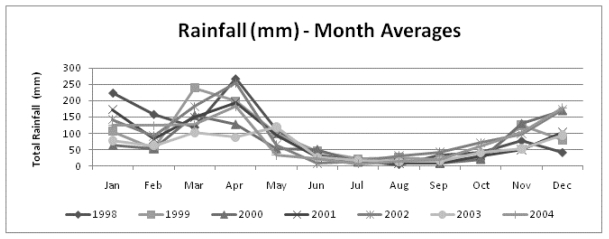
Seasonal distribution of rainfall in Tanzania: Monthly averages (mm).

The rather limited reliability of the econometric analyses due to the reduced number of observations (7 years) in the datasets should be acknowledged in advance. The dataset containing data aggregated at the monthly level allowed the observation of any seasonal trend in cholera cases and climate variables that cannot be observed with the annual data. Looking at the data, it was observed that a seasonal pattern seems to exist between June and October when lower minimum/maximum temperatures and lower total rainfall coincide with lower cases of cholera (Figure 1 and Figure 2).

### 2.2. Health Impact Assessment

In order to analyze the magnitude of the burden of cholera attributable to climate change, it is useful to decide on the different scenarios that the analysis will be based on. For this purpose, climate-change predictions for the specific locality, as well as population growth and economic development, will need to be considered. The approach used in this study follows the methodological framework suggested by Chiabai *et al*. [34], who estimated the additional costs of planned adaptation in the health sector in India for 2030. Two scenarios are suggested. 

*Scenario C_0_*, for 2030, is the baseline scenario and does not include climate change. The scenario is based on the baseline projections of Mathers and Loncar [35]. These projections include estimates for diarrheal diseases in 2030 for the main world regions, based on WHO estimates for 2002. The estimates are based on past correlations between increased growth and mortality trends for specific illnesses. The estimates make two main assumptions. First, economic development in the form of increasing GDP and other socio-economic developments are expected to have a positive influence on the performance of the health sector. Therefore, the relative burden of disease in 2030 is expected to decrease considerably when compared with current levels due to economic development, with improvements in the performance of the health sector and increased coverage of water and sanitation (basic preventive adaptation measures). Secondly, developing countries are expected to have constant growth without crises or negative impacts on economic development. Mathers and Loncar [35] take into account GDP per capita, years of schooling by adults and the impact of technology on population health measured with a time variable. Population projections for 2030 are included based on UN-projected population figures [36], taking into account the fertility rate and immigration. 

As the projections for diarrheal diseases are not released at country levels but are only available at the regional level, the initial step was to downscale the projected number of deaths at the country level. This was done using the projections of Mathers and Loncar [35] of total deaths from diarrheal diseases to calculate the percentage contribution of each country in Sub-Saharan Africa to the projected regional burden of disease. As the projections are provided only for diarrheal diseases as an aggregate, the next step was to calculate the corresponding number of deaths for cholera. For this purpose the proportion of deaths caused by cholera with respect to diarrheal deaths in Tanzania was calculated based on *WHO Weekly Epidemiological Reports* 2000–2010 [37]. Next, the number of cholera cases was estimated based on the derived estimate of deaths using the average case fatality rate (CFR) of 3.18 percent for cholera deaths in Tanzania, which was also based on the *WHO Weekly Epidemiological Reports* 2000–2010. The results from the econometric analysis described in the next section suggest a CFR equal to 0.078. However, the dataset available for this estimation comprised only 15 observations, which compromises the reliability of this estimate. Therefore, the average CFR for cholera in Tanzania provided by the WHO was used, which is also a more conservative estimate (0.0318). 

Lastly, to supplement the estimated deaths and cases, a measure of population health has been estimated by combining data on mortality and non-fatal health outcomes into a single figure. For this purpose, Disability-Adjusted Life-Years (DALY) was applied. Several other measures, besides DALY, have been devised in the literature, including the Quality-Adjusted Life Year (QALY), Disability-Adjusted Life Expectancy (DALE) and the Healthy Life Year (HeaLY) [38,39,40,41]. The benefits and challenges of the various measures have been examined [42,43,44,45]. It should nonetheless be noted that the DALY measure has been criticized because it does not use different weights for sexes or different age weights, does not discount future years lost, and, lastly, it does not use severity weighting of disabilities. However, since DALY has been the most widely used measure and is often used as an index in the comparison of health impacts in studies of disease, the DALY metric has been used for the purpose of this study. 

DALY measures disease burden and combines years of life lost (YLL) from premature deaths with years of life lived with disabilities (YLD). *YLL* is estimated as *YLL* = *N_D_* × *E_X_* where *N_D_* is number of deaths, and *E_X_* is life expectancy at age of death. A life expectancy at birth of 63.8 years (both sexes combined) by 2030 based on the UN World Population Prospects [35] is used. To allow for the estimate of life expectancy at age of death, it is necessary to obtain the age distribution of cholera deaths. For this purpose, WHO estimates of the distribution of diarrheal diseases between age groups in Sub-Saharan Africa are used, assuming that this distribution is the same for Tanzania [14]. Here, a limitation of this study must be acknowledged given the availability of data, since it has not been possible to retrieve the age distribution specifically for cholera deaths in Tanzania as a whole, nor on a regional basis. Nonetheless, the estimates include cholera (and other diarrheal diseases). It may, however, have an impact on the results, since there may be an overweight of the share of deaths among children under 5 years of age, which may not be the same for cholera as it is for diarrhea. *YLD* is estimated as *YLD* = *I* × *DW* × *L*, where I is the total number of cases, *DW* is disability weight, and *L* is the duration of each cholera episode. *YLD* estimates are limited to loss of health experienced by individuals, and do not take into account other aspects of the quality of life or well-being, or the impacts of one individual’s health condition on other people. Disability weight captures valuation of time lived in a non-optimal health state viewed from societal preferences. The disability weight ranges between 0 (health condition is equivalent to full health) and 1 (health condition is equivalent to death) and quantifies judgments about the overall levels of health associated with different health states, not judgments on the relative values of lives lived, persons, overall well-being, quality of life or utility. In estimating *YLD* a disability weight of 0.15 is used. This is equivalent to that used by the WHO [46], which is intended to reflect average global valuations. Again, the data availability limits the estimates to use the weight provided for diarrheal diseases as an aggregate. Information on the average duration of a cholera episode (5 days) was obtained from the Tanzanian Ministry of Health. In the estimates, it was assumed that age-sex-specific incidence ratios would remain constant into the future, as would average durations and disability weights.

The second scenario, *Scenario C_1_*, builds on *Scenario C_0_* and estimates the number of cholera cases, deaths and DALYs in the light of projected climate-change impacts. The estimates are produced by taking into account the relative risk of contracting cholera projected for 2030, which represents the relative change in the dependent variable given a one-unit change in the explanatory variable. The relative risk applied here is based on an econometric analysis of the relationship between climate variability and cases of cholera as described in the next section. The relative risks are applied to the projected number of cases calculated in *Scenario C_0_*. The difference between the estimates derived in the two scenarios (with and without climate change) represents the burden of disease attributable to climate change. The calculations for *Scenario C_1_* assume a 1 and 2 degree Celsius temperature increase respectively, based on the IPCC [3], A1B middle scenario, temperature predictions for Tanzania for 2030.

Calculations of the burden of disease attributable to climate change are based on the Impact Fraction suggested by McMichael *et al.* [5]. The Impact Fraction is the fraction of the total disease burden attributable to climate change, and is calculated by combining the proportion of population exposed with the relative risks:


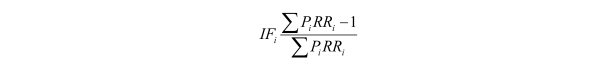


where IF_i_ is defined as the impact fraction attributable to climate change for disease i; P_i_ is the proportion of the population exposed to the disease; and RR_i_ is the relative risk of the disease under different climate scenarios.

#### Assessing the Relationship Between Climate Variability and Cholera Cases 

In order to investigate trends in the seasonal pattern, regression analyses, using Stata/SE version 10.1 for Windows, were conducted where a dummy variable, ‘drought’, equals 1 between June and October and zero at other times. A time-trend variable was also used to capture the possible socio-economic effects on cholera cases that were not available at the month level. For example, it seems reasonable to assume that the population, the percentage of the population with access to safe water and sanitation, education levels and income all grew over time between 1998 and 2004.

In order to investigate the potential relationship between climate variability and cases of cholera to identify the relative risk, Poisson regression analysis was initially used (similar to others in the field; see e.g., Singh *et al.* [13]; Kuhn *et al.* [47]; Tango [48]). A Poisson regression is used to fit models of the number of occurrences or counts of an event (dependent variable) assuming that they follow a Poisson distribution. However, the Poisson regression analysis requires that the data present conditional variance equal to the conditional mean. Therefore, we performed log-likelihood (goodness of fit) tests after all Poisson models initially estimated and confirmed that the required non-dispersion assumption is violated. In other words, the count data present over dispersion, invalidating the use of Poisson models, and alternative models were required for the analysis, such as the negative binomial model [we do not present the Poisson models’ results in the annex since they were not used in our calculations due to the overdispersion problem. Instead, we present in the annex our preferred negative binomial models based on the usual tests (e.g., log-likelihood test; Wald-test; Akaike test; BIC)]. A negative binomial regression model estimates the expected value of the dependent variable as a log-linear function of a set of independent variables and regression parameters, while correcting for data over dispersion [49]. The specifications used in the negative binomial models are of the general form:


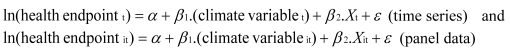


where health endpoint is the incidence of cholera cases or deaths in period t (month or year); climate variable represents rainfall or temperature; and X is a vector of socioeconomic factors that may explain the health endpoint (population growth, GDP, water and sanitation coverage, literacy rate and cassava production per capita). For each dataset, different models have been tested with specifications combining all the available explanatory variables. The preferred models were chosen according to the level of significance of regressors in each model and the goodness-of-fit of each model (log-likelihood; Wald chi-squared; and Akaike and Bayesian information criteria). Details of the preferred models are given in Annex 1. A time-trend variable was integrated into the model to capture the effect of other socioeconomic variables that potentially influence cholera cases and that were absent from this dataset.

### 2.3. Costs of Cholera Attributable to Climate Change

In the context of assessing the total costs of health impacts attributable to climate change, several measures will need to be taken into account. These include preventive and reactive adaptation measures, in addition to the costs of residual damages. In the case of cholera (and many other health issues), it is far from realistic to assume that preventive adaptation measures can reduce the risk of disease equivalent to the additional risk of disease due to climate change. Hence some costs are expected to be allocated to reactive adaptation measures in terms of cholera cases continuing to be treated after preventive adaptation measures have been put in place. Nevertheless, only a certain proportion of health impacts are assumed to be reduced with adaptation measures, and the costs of residual damages in terms of morbidity and mortality which cannot be avoided with adaptation measures need to be included in the total costs of the impacts. All costs are calculated at constant 2006 US dollar rates.

#### 2.3.1. Assessment of Preventive Adaptation Measures

Preventive adaptation measures, which in the case of cholera are thought of mainly in the form of hygiene, water and sanitation programmes [19], are assumed to be initiated and implemented in line with general socio-economic development in Tanzania and the national goals of near-universal water and sanitation coverage by 2025 [50]. An integrated part of Tanzania’s development strategy is structural developments which have been initiated in the water sector [50]. These included the Water Sector Development Programme (WSDP) which started in July 2007. WSDP is a twenty year nationwide programme for improving the provision of water supply and sanitation services. Moreover, the programme also includes initiatives on awareness raising campaigns and education to stakeholders on how to reduce incidences of disease. These programmes are established outside the health sector, but nevertheless bring large health benefits along with other domestic and productivity benefits in terms of time saved for water collection, increased time for education and leisure activities, and benefits to agricultural and other income-generating activities [51]. Because the preventive measures are assumed to be implemented along with economic development in Tanzania, the costs of prevention are not included in the total estimates of adaptation costs measures in this study. However, to give an indication of the costs of prevention, a recent study [20] found household-based chlorination to be the most cost-effective intervention, with an annual cost of US $59 per DALY averted. 

#### 2.3.2. Reactive Measures

The cholera cases that remain after preventive adaptation measures have been introduced are treated with reactive measures. First, information on cost estimates and the management of a cholera patient admitted to a hospital was obtained from the Tanzanian Ministry of Health in Dar es Salaam. Cholera patients are not treated as outpatients but isolated in a special cholera ward or unit during treatment. Treatment on average takes five days per case and includes infusions, oral rehydration salt, and antiseptics. In addition to treating the cholera patient, people who have had contact with cholera patients are placed under surveillance for five days and given preventive treatment or therapy as outpatients. The total unit cost per case of managing a cholera-admitted, patient including treatment, cost of bed, personnel and outpatient surveillance, is, according to the Ministry of Health, US $98. In the cost estimates in this study, it is assumed that all cases that remain after the introduction of preventive measures are treated with reactive measures. The additional costs of reactive adaptation measures are therefore calculated by multiplying the number of cases attributable to climate change (calculated for *Scenario C_1_*) with the unit costs per case treated.

#### 2.3.3. Lost Short-Term Productivity

Ideally, the cost estimates of reactive adaptation measures do not represent the total value of the additional costs of climate change, since loss of productivity should be included in estimating the direct total costs of illness (costs of treatment, plus loss of productivity). Therefore, the costs of morbidity include productivity losses that remain after the introduction of preventive adaptation measures. These losses are calculated from daily wages multiplied by days out of work as a result of being sick. For this purpose, the average weighted wage for a person aged fifteen and above is based on the employment figures given in the Tanzanian Integrated Labor Force Survey 2006 [52]. The weighted wage rate includes wage rates for the self-employed (USD 56/month), formal employees (USD 72/month) and the unemployed (USD 37/month). The estimate for the unemployed is based on the assumption that even those who are not formally employed will still contribute to economic activities. Therefore a wage corresponding to an agricultural worker’s wage is used as a proxy to estimate productivity losses for the unemployed proportion of the population. Also, for the cholera cases among children, it is most likely that an adult will spend time away from work to take care of the sick child and hence withdraw time from productive activities. It is therefore assumed in the estimates that all cases of cholera result in a productivity loss equivalent to the weighted daily wage rate multiplied by five working days.

#### 2.3.4. Loss of Lives

To account for the loss of lives attributable to climate change, a GDP-adjusted Value of Statistical Life (VOSL) method has been used. Broadly VOSL measures individual willingness to pay to reduce the risk of death and it has been used widely in environmental economics to value mortality impacts [53]. The VOSL estimate for Tanzania is calculated based on a VOSL for the US of USD 4 million and adjusted to Tanzania using the ratio of real per capita GDP and taking purchasing power into account. The GDP estimates were obtained from the World Development Report database [54]. Based on this, a VOSL for Tanzania amounting to USD 32,958 is estimated. The total cost of cholera deaths attributable to climate change henceforth can be measured from *Cost_d_* = *deaths*_2030*cc*_ × *VOSL*, where *Cost_d_* is the total cost of cholera deaths attributable to climate change, and *deaths_2030cc_* is the number of deaths attributable to climate change.

#### 2.3.5. Total Cost of Cholera

Finally, the total economic costs of cholera attributable to climate change are estimated by totaling the costs of reactive adaptation and the costs of impacts remaining after the introduction of preventive adaptation measures. The costs of impacts are estimated from cases (productivity losses) and deaths. As previously mentioned, the costs of preventive measures are not included in the calculations of total costs of cholera attributable to climate change, since these cost measures are assumed to be implemented in line with Tanzania’s national development objectives. The GDP share of total cost in Tanzania by 2030 has been calculated on the basis of GDP projections for annual GDP growth rates for Sub-Saharan Africa [55].

## 3. Results

### 3.1. Health Impact Assessment

The pair-wise correlation analysis showed that cholera cases are positively correlated with minimum temperature, maximum temperature and their one-month lags (all statistically significant). The linear correlation between cholera cases and total rainfall was not significant. This result suggests that cholera cases in Tanzania might be better explained by temperatures than by rainfall. The results using the dataset gathered per month (Annex 1, Table A1) showed a positive and significant association between cholera cases and temperature, and a negative and significant association between cholera cases and the drought season, reflecting the observed reduction of cholera cases between June and October (Figure 1). The signs of the estimated coefficients for both explanatory variables (rainfall and temperature) were as expected. Seasonality was dealt with in the analysis via the introduction of a dummy variable indicating the period (months) of less or more rainfall. The variable “drought” resulted indeed in statistical significance in the model using monthly data and was kept in the final model. The analysis also dealt with seasonality by testing one-period lags of all climate variables in all models, which did not result significant. The negative sign of the coefficient of variable “drought” confirms that cholera cases decrease in months with less rainfall, as expected. Regarding the test of one-period lags of climate variables, the significance level of the coefficients showed no statistical relationship among cholera cases and temperature and rainfall observed in the previous month of the cholera occurrence. 

The risk ratio was estimated to be equal to 1.29 using the monthly data set. In other words, an increase in temperature equal to 1 degree Celsius would increase the relative risk for cholera cases in Tanzania by 29 percent. Using the annual data for all Tanzania’s regions (the panel of regions), temperature also explained cholera cases better (Annex 1, Table A2). In this model, an increase in temperature equal to 1 degree Celsius would increase the relative risk for cholera cases in Tanzania by 15 percent. No statistically significant model associating total annual cholera cases in Tanzania with annual average temperatures or rainfall could be obtained using the annual dataset for the country as a whole. The results of the computed cases and deaths attributable to climate change for 2030 are presented in Table 2 for each of the scenarios. In these, the CFR provided by WHO were assumed, as mentioned earlier, rather than the estimate from the modeling in the current study (Annex 1, Table A4). The projections are estimated for a lower boundary and an upper boundary. The lower boundary represents the results from the annual dataset, with an increase in the relative risk of 15 percent for a 1 degree Celsius increase in temperature. The upper boundary represents the results from the monthly dataset, with an increase in relative risk of 29 percent for a 1 degree Celsius increase in temperature. The table also includes estimates of DALYs. 

**Table 2:** Estimated burden of cholera disease attributable to climate change in 2030.

	Scenario C_0_ (2030)	Scenario C_1 _(1 °C 2030)*		Scenario C_1_ (2 °C 2030) *	
		Lower	Upper	Lower	Upper
cholera cases	369,783	425,250	477,020	489,038	615,356
additional cases		55,467	107,237	119,255	245,573
cholera deaths	11,759	13,523	15,169	15,551	19,568
additional deaths		1,764	3,410	3,792	7,809
DALYs	555,312	716,358	638,613	734,405	924,101
additional DALYs		83,302	161,046	179,094	368,790

* cholera cases and deaths in Scenario C_0_ (2030) refers to a scenario without climate change but taking into account economic growth only, while the additional number of cases in Scenario C_1_ (1 °C 2030 ) and Scenario C_1_ (2 °C 2030) are those specifically related to climate change.

The total number of cases is expected to increase from the baseline of 369,783 cases up to as many as 615,356 cases, with an additional 245,573 cases for the upper boundary scenario, given a 2 degree Celsius temperature increase by year 2030. For the more moderate scenario with only 1 degree Celsius increase in temperature, still with the upper boundary scenario, the additional number of cases will be 107,237. For the most moderate scenario, namely lower boundary and 1 degree Celsius increase in temperature, the additional number of cases attributed to climate change will be 55,467. With regard to deaths, the numbers will increase from the baseline of 11,759 deaths to 19,568 for the upper boundary scenario with a 2 degree Celsius temperature increase. This is equal to an additional 7,809 deaths attributable to climate change. For the most moderate scenario, the additional number of deaths is limited to 1,764. 

### 3.2. Costs of Cholera Attributable to Climate Change

The results of the cost estimates (Table 3) shows that the annual costs of reactive adaptation measures for managing climate change-attributable cases of cholera in 2030 range from USD 5.5 million to USD 10.5 million in the optimistic scenario with a 1 degree Celsius increase in temperatures, and from USD 11.7 million to USD 24 million in the pessimistic scenario with a 2 degree Celsius increase in temperatures. Comparison of the reactive adaptation costs in the two scenarios shows that a cost reduction of 44 to 47 percent can be achieved if the temperature increase is kept at 1 degree Celsius. However, these cost estimates are based on the assumptions that preventive adaptation measures are introduced, such as infrastructure for water and sanitation, and that Tanzania meets its national goals of nearly universal water and sanitation coverage by year 2025. If adequate preventive adaptation measures are not sufficiently introduced, the burden of disease and the costs of additional cases of cholera and related deaths would be much higher than the indicated estimates in Table 3. 

**Table 3:** Annual costs of cholera attributable to climate change by 2030 in Tanzania (USD).

Scenario	Cost of reactive measures	Productivity losses	Loss of lives	Total costs	Total cost (GDP, %)
Scenario C_1 _(1 °C 2030)					
Lower	5,430,815	504,970	58,133,424	**64,069,209**	0.32
Upper	10,499,575	976,276	112,391,286	**123,867,137**	0.61
Scenario C_1 _(2 °C 2030)					
Lower	11,676,252	1,085,686	124,986,861	**137,748,799**	0.68
Upper	24,044,027	2,235,671	257,376,045	**283,655,743**	1.40

Table 3 includes the results of productivity losses for each scenario. The productivity losses account for approximately 11 to 13 percent of the total cost of illness (cost of reactive adaptation + productivity losses). This corresponds roughly to previous estimates for malaria in South Africa, which show loss of productivity to account for 8 percent of the cost of illness [56]. A large share of the costs (90 percent) is related to loss of lives, though even without considering the value of lost lives, the cost of illness (reactive measures + productivity losses) accounts for 0.03 to 0.13 percent of GDP. The estimates of the GDP share of total costs of cholera attributable to climate change are shown in the last column of Table 3. The upper boundary estimate for the 1 degree Celsius scenario and the lower boundary estimate for the 2 degree Celsius scenario lie at around 0.6 percent of GDP. This is a considerable share of GDP, bearing in mind that the full health costs of climate change would be much larger if other health variables affected by climate change, such as other water-borne diseases besides cholera (e.g., diarrhea, typhoid), malnutrition, food-borne (e.g., Salmonella) and vector-borne diseases (e.g., malaria, dengue), were taken into account. 

## 4. Discussion

### 4.1. Health Impact Assessment

There are few detailed studies of adaptation costs in the health sector, and this study is the first to link cases of cholera to environmental and socioeconomic factors in predicting climate change impacts to cholera in Tanzania and assessing the related costs. Unlike earlier studies costing adaptation, this study does not keep the baseline incidence of disease fixed at current levels. Instead it incorporates a future baseline disease burden, which implies a reduction in the incidence rates that may be primarily attributed to economic growth and developments that the health sector would undergo in the future. However, accurate cost estimates require robust impact assessments and, given the lack of consistent long-term datasets, the reliability of the econometric analyses is to some extent limited. It is likely that, with more time-specific data available on health and climate variables, the results could show even stronger impacts. 

In addition, the impacts of climate variability on the burden of disease in the form of cholera are complex and dependent on a number of risk factors from local socio-environmental conditions. For a waterborne disease, the results presented in this paper may seem high, with a 15 to 29 percent increase in the relative risk per degree Celsius, in comparison to the 5 percent increase as predicted by WHO [14] for Sub-Saharan Africa in their GBD study. However, this difference could be could be explained by sub-regional differences. Additionally, Wang *et al.* [57] predict an increase in diarrheal incidences of 0.6 percentage point for a 1 degree Celsius increase in temperatures for South Arica. The estimates presented in this paper predict an increase in cholera incidences of 0.05 to 0.1 percentage point for a 1 degree Celsius increase in temperatures for Tanzania, so the results do not seem unreasonably high, considering that cholera is included as a diarrheal disease in the study by Wang *et al.* [57]. The results in this study conform to what would be expected given previous evidence on linkages between environmental risk factors and cholera in Africa [6,7,8,9], adding to the existing evidence of the implications of climate change for cholera. 

### 4.2. Costs of Cholera Attributable to Climate Change

The cost estimates used for reactive adaptation are based on unpublished data from the Tanzanian Ministry of Health. These costs are considerably higher than the costs of treating diarrhea since diarrheal patients can be treated as outpatients, while treatment of cholera has to include hospitalization for an average of five days per case, as well as the cost of surveillance of other people than the patient. In Zambia, average inpatient costs for diarrhea are estimated to by USD 78 per bed day [58], while for Tanzania inpatient costs are estimated in the range of USD 3.40–11.86 per day [59]. The latter figures correspond well to the cost figures provided by the Tanzanian Ministry of Health of USD 98 for five days of hospitalization. For South Africa, the reactive costs of diarrheal cases attributed to climate change by year 2020 are estimated to be equivalent to a 0.2–0.52 percent share of GDP [57]. On this basis, the reactive costs of additional cases of cholera in Tanzania by 2030, which are in the range of 0.03–0.12 percent share of GDP, do not seem unreasonably high. 

In estimating total costs, the costs of preventive measures were not included, since it was assumed that these would be implemented along with general socio-economic development and according to Tanzania’s national goals of near-universal water and sanitation coverage by 2025. Accordingly, improved sanitation and hygiene facilities are vital components of the planning and provision of water supply services [50]. However, as already mentioned in the results section, if adequate preventive adaptation measures are not sufficiently introduced, the burden of disease and the related costs will increase considerably. The recent National Strategy for Growth and Reduction of Poverty II [60] for Tanzania nevertheless reports that access to rural water supply services increased from 55 percent in 2005 to 58.7 percent in 2009, and from 74 percent to 84 percent in urban areas, and key structural developments have been and are being initiated in the water and sanitation sector. Hence the assumption that Tanzania will meet its goal by 2025 may not be unrealistic.

Another problem arising from the cost estimates is the issue of valuing costs that take the form of increases in mortality [24]. This implies lost life years, which in turn requires a value to be attached to a life year or a life lost. This has stimulated discussions of the methods used in making such estimates, where the use of different values for a life year according to the country in which the person lives has raised serious problems when assuming a lower value of life in developing countries compared to industrialized countries. Some have argued for using different values for different countries, which are in proportion to real per capita GDP [61], while others have used the same value for all lives saved, irrespective of the country in which the person is resident [62]. In addition, it has been argued that the willingness to pay for improved health may only include the welfare impacts due to illness and can thus be criticized for discriminating against the poor, who have less ability to pay due to their relatively low incomes. Nevertheless, the GDP-adjusted VOSLs which are included in the estimate of total costs of cholera attributable to climate change definitely has an impact on the overall result since the loss of lives therefore accounts for 90 percent of total costs. One aspect which was not considered during the cost calculations were the impacts—or benefits—from autonomous adaptation at the individual, household or even community levels, where adaptation would occur without public intervention given the policies or project being implemented. Another feature which was not considered is the distributional impacts of adaptation measures. The distribution of benefits from both preventive and reactive adaptation measures is often challenged, and people with low bargaining power tend to be the last to receive benefits, if any. Benefits in this case would be reduced vulnerability to cholera from improved water access and sources, as well as medical treatment in cases of illness. 

## 5. Concluding Remarks

The results presented in this article, despite their limitations, increase our understanding of the implications of climate change on health impacts in Tanzania, and provide indications of cost implications imposed by the additional burden of cholera.

Integrating both climate variables and socioeconomic variables in one model confirms that conditions of human health are influenced by many factors and cannot be addressed in isolation. The results of these effects in relation to the impacts of climate change suggest that it would be highly beneficial to improve socioeconomic indicators, including access to water and sanitation, even more quickly than originally planned. The existing time frame for water and sanitation programmes is challenged, with considerable benefits to be gained from more rapid implementation than originally planned. This also includes health education, levels of health service systems and disease surveillance, in addition to measures for early diagnosis and prompt treatment, and the control or prevention of epidemics. Such health-based measures should be viewed in the context of broader development, where the main means to adapt is improvements in general living standards. There is unquestionably a wide array of other benefits for improving performance on these indicators, since they influence a number of other development objectives such as nutrition and education. 

The estimates, based on assumptions of climate-change projections, suggest that the cholera health cost of increased temperatures of 1 to 2 degree Celsius by 2030 will be in the range of 0.03 to 0.12 percent of GDP for treatment costs alone (cost of reactive adaptation), while total additional cost attributable to climate change, including productivity losses and the value of lost lives, averages out at around 0.75 percent, but may be as much as 1.4 percent of GDP by 2030 for the upper boundary. The magnitude of these cost estimates is substantial, and considerably higher than the current budgets allocated for diarrheal diseases in most developing countries. Thus budgetary increases will be needed if treatment is to be provided to deal with the additional burden of disease attributable to climate-change which cannot be avoided through preventive adaptation measures.

The current study has several strengths compared to previous literature on the implications of climate change and the costs of adaptation in the health sector. Compared to most estimates, which are made on regional levels, the scope of assessment has been on the national level, offering interesting insights for policy-makers in evaluating potential adaptation measures at more local and national levels. The cost estimates provide useful information which can feed into both national and international debates on financing and planning adaptation. In addition, the estimates take into account expected future economic development in Tanzania, which includes a strengthening of the health system and hence has an impact on the projections of incidence.

The implications of the results for future studies suggest that climate change will cause large additional economic burdens for both societies and households. Consequently, it is vital to quantify the burden of disease attributable to climate change at the national and local levels as opposed to the regional levels, since the vulnerability of human health to climate change in terms of exposure (environmental variables) and capacity to cope and adapt (socioeconomic variables) may vary considerably between time and place. Therefore more and improved projections of future risks are necessary for local (national) decision-making, in addition to making further efforts to refine the national costs of preventive and reactive adaptation measures in the health sector. 

## Annex 1: Econometric Results

**Table A1:** Negative binomial model—Monthly data: Cholera cases.

	Coefficient	Robust Std.Err.	P > |z|	IRR	Robust Std.Err.
Constant	-18.681 ^***^	3.2578	0.000	–	–
Max Temperature	0.2557 ^**^	0.1109	0.021	1.291	0.1432
Drought	-0.5952 ^*^	0.3347	0.075	0.5514	0.1846
Trend	0.0068	0.0054	0.217	1.006	0.0054
Observations	84				
Log pseudolikelihood	-592.07138				

Notes: Negative binomial regression—dependent variable is number of cholera cases per month—exposure group is population. *** significant at 1%; ** significant at 5%; * significant at 10%.

**Table A2:** Negative binomial model—Annual data (panel of regions): Cholera cases.

	Coefficient	Robust Std.Err.	P > |z|	IRR	Robust Std.Err.
Max Temperature	0.1412 ^**^	0.0636	0.027	1.1516	0.0733
Constant	-5.4503 ^***^	1.8444	0.003	–	–
Observations	119				
Log pseudolikelihood	-527.82738				

Note: Random-effects Negarive binomial regression—dependent variable is number of cholera cases per year per region. *** significant at 1%; ** significant at 5%; * significant at 10%.

**Table A3:** Negative binomial model—Annual data: Cholera deaths and income.

	Coefficient	Robust Std.Err.	P > |z|	95% confidence interval	
Constant	-10.6916 ^***^	0.1270	0.000	-10.9406	-10.4427
Cholera cases	0.00016^ ***^	0.000018	0.000	0.00012	0.0002
Real GDP per capita	-0.0028 ^***^	0.0002	0.000	-0.0033	-0.0024
Observations	28				
Log pseudolikelihood	-161.6423				

Notes: Negative binomial regression—dependent variable is number of cholera deaths per year. *** significant at 1%; ** significant at 5%; * significant at 10%.

**Table A4:** Negative binomial model—Annual data: Cholera deaths and water cover.

	Coefficient	Robust Std.Err.	P > |z|	95% confidence interval	
Constant	-5.6968 ^***^	0.8729	0.000	-7.4076	-3.9859
Cholera cases	0.0001 ^***^	0.00001	0.000	0.0001	0.0002
Water cover	-12.5607 ^***^	1.5875	0.000	-15.6721	-9.4492
Observations	15				
Log pseudolikelihood	-84.2818				

Notes: Negative binomial regression—dependent variable is number of cholera deaths per year. *** significant at 1%; ** significant at 5%; * significant at 10%.
